# Efficient Synthesis of Glucovanillin and Elucidation of Its Molecular Mechanisms in Ameliorating T2DM via Core Target Modulation and α-Glucosidase Inhibition

**DOI:** 10.3390/molecules31132228

**Published:** 2026-06-24

**Authors:** Huanyu Zhang, Weiqian Zhang, Fangya Li, Xinyao Lu, Yuping Yan, Dan Zhang

**Affiliations:** 1 Traditional Chinese Medicine Processing Technology Innovation Center of Hebei Province, Hebei University of Chinese Medicine, Shijiazhuang 050200, China; zhanghychn@163.com (H.Z.); yjs20252134@hebcm.edu.cn (W.Z.); yjs20242112@hebcm.edu.cn (X.L.); 2 School of Mathematical Sciences, Hebei Normal University, Shijiazhuang 050024, China; lifangya@hebtu.edu.cn

**Keywords:** enzymatic synthesis, type 2 diabetes mellitus, network pharmacology, machine learning, molecular dynamics simulation

## Abstract

This study focuses on the synthesis of glucovanillin mediated by UGT109A1 and its mechanism against Type 2 Diabetes Mellitus (T2DM). Recombinant UGT109A1 successfully synthesized glucovanillin from vanillin using UDP-Glc as the sugar donor. Through network pharmacology, 140 potential targets were identified. Seven key targets were further screened using LASSO and SVM-RFE algorithms. Among these, SLC5A1 and ADK showed strong diagnostic potential, with AUC values ranging from 0.85 to 0.89. Immune infiltration analysis linked these core targets to M2 macrophages. Single-cell transcriptomics revealed that ADK is widely expressed but enriched in B cells, while TLR9 is confined to plasmacytoid dendritic cells (pDCs). Cell-to-cell communication analysis identified a pDC-to-B cell signaling axis. In vitro assays demonstrated that glucovanillin exhibits concentration-dependent inhibitory activity against α-glucosidase with moderate potency, with an IC_50_ of 413.84 ± 12.80 μM. Molecular docking, 200 ns molecular dynamics simulations (MD), and MM/PBSA calculations showed that glucovanillin binds more strongly to α-glucosidase (−7.4 kcal/mol) than vanillin (−5.4 kcal/mol). Therefore, the glycosylation mediated by UGT109A1 enhanced the bioactivity and targeting specificity of vanillin. In summary, glucovanillin exerts anti-T2DM effects through a dual mechanism involving α-glucosidase inhibition and regulation of key targets, making it a promising lead compound for T2DM treatment.

## 1. Introduction

Type 2 Diabetes Mellitus (T2DM) is one of the most prevalent and rapidly increasing metabolic diseases worldwide [1]. It constitutes a significant and escalating public health challenge with a profound impact on human health. T2DM mainly affects adults, accounting for approximately 90% of all diabetes cases [2]. Its characteristic features include insulin resistance and the gradual decline of pancreatic β-cell function. If left unmanaged, chronic T2DM can lead to a series of serious complications, including cardiovascular diseases [3], neuropathies [4], and retinopathy [5], all of which will significantly reduce the quality of life of patients.

Within the intricate blood glucose regulation network of T2DM, postprandial hyperglycemia has become a key driver of disease progression. *α*-Glucosidase is a key enzyme regulating this condition and has become a focus of efforts to discover new therapeutic drugs [6]. Presently, *α*-glucosidase inhibitors, such as acarbose, are widely used in clinical practice and can effectively reduce postprandial blood sugar levels [7]. However, these drugs lack specificity in their mechanism of action, and long-term use may cause adverse reactions, including insomnia, headaches, and constipation [8]. This clinical limitation has catalyzed the ongoing quest for the development of new-generation inhibitors, which are not only more efficient but also exhibit reduced toxicity. This represents a highly promising approach for improving the treatment strategies for T2DM.

Natural products (NPs) offer significant advantages in the development of therapeutic agents for T2DM, owing to their diverse structures, extensive biological activities, and relatively low toxicity [9,10]. Vanillin is a phenolic compound abundantly present in plants such as vanilla beans, cloves, and cinnamon. Recently, it has garnered attention due to its significant pharmacological effects. Empirical studies demonstrate that vanillin acts as a potent *α*-glucosidase inhibitor, with an IC_50_ value of 28.34 ± 0.89 mg/mL [11]. Furthermore, vanillin, in synergy with rice extract, has shown promising efficacy in regulating the activity of *α*-glucosidase [12]. However, due to inherent limitations, such as poor water solubility, suboptimal bioavailability, and insufficient target binding concentration, the clinical application of vanillin has been hindered. A key strategy to overcome these limitations is to improve its pharmacokinetic and pharmacodynamic properties through structural modification.

Glycosylation is a classical strategy for the structural optimization of NPs, involving the introduction of sugar moieties onto the active molecular framework [13]. This process not only significantly enhances the water solubility and bioavailability of the compound, but also augments its specificity and binding affinity towards the target enzymes. For instance, the addition of two glucose units to the aglycone of protopanaxadiol (PPD) effectively enhances the anticancer activity of ginsenoside Rg3 [14]. In enzymatic glycosylation reactions, UDP-glucosyltransferases (UGTs) play a crucial role as key catalytic enzymes, transferring sugar moieties from donors to various compounds, such as phenolics [15], terpenoids [16], and flavonoids [17], at active sites like hydroxyl and carboxyl groups, resulting in more active glycoside derivatives [18,19]. Among these, UGT109A1, an important member of the UGT superfamily, was initially isolated and identified from the model strain *Bacillus subtilis* and is known for its broad substrate adaptability and high catalytic efficiency [20]. This enzyme has already been successfully employed in the glycosylation of NPs like ginsenosides and glycyrrhetinic acid [21,22]. However, previous studies on UGT109A1 have primarily focused on triterpenoid substrates for producing unnatural glycosides with enhanced anticancer activities. To the best of our knowledge, the substrate spectrum of UGT109A1 has not yet been extended to small phenolic compounds such as vanillin. In contrast to plant-derived UGTs (UGT71C2 and VpUGT72U1) previously reported for vanillin glycosylation [23,24], UGT109A1 offers potential advantages in regioselectivity and catalytic efficiency due to its bacterial origin. To date, no reports have utilized UGT109A1 to catalyze the synthesis of glucovanillin. The feasibility of its synthesis and the anti-T2DM activity of the product remain to be systematically validated.

Based on the aforementioned research background, this study employs UGT109A1 as the catalytic enzyme to construct an enzymatic synthesis system for glucovanillin, and verifies the product structure through LC-MS/MS technology. Subsequently, using network pharmacology, machine learning, and immune infiltration analysis, the core targets of glucovanillin in improving T2DM are systematically screened, and its action pathways and immune regulatory mechanisms are elucidated. Finally, molecular docking, 200 ns MD simulations, and MM/PBSA free energy calculations are employed to verify the binding affinity and stability of glucovanillin with *α*-glucosidase. This study (a) is the first to use UGT109A1 to catalyze the synthesis of glucovanillin, providing a novel method for the green and efficient preparation of this compound; (b) integrates multidisciplinary technologies to systematically reveal the mechanisms of glucovanillin’s anti-T2DM effects at the molecular, cellular, and immune levels, offering theoretical support for its clinical application; (c) clarifies the optimization effect of glycosylation modification on the *α*-glucosidase inhibitory activity of vanillin, providing insights for the structural modification of NPs. Therefore, the findings of this study hold promise for providing novel candidate drugs for T2DM treatment and advancing the application of NP glycosylation technology in the pharmaceutical field.

## 2. Results and Discussion

### 2.1. Enzymatic Synthesis of Glucovanillin with UGT109A1

**Figure 1:**
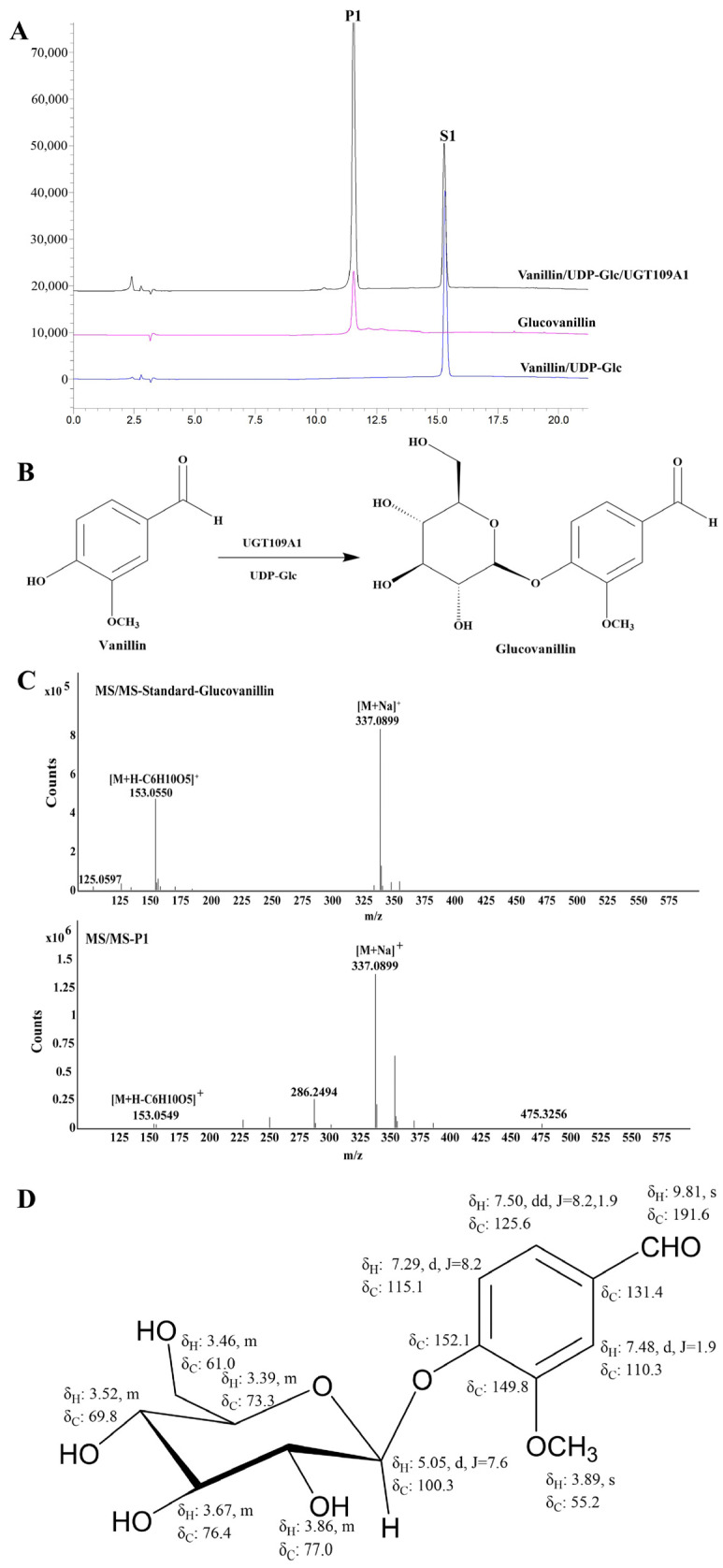
Analysis of UGT109A1-catalyzed glycosylation products using vanillin as the substrate. (**A**) HPLC assessment of UGT109A1-mediated enzymatic reactions, with chromatographic data recorded at 328 nm; (**B**) The glycosylation of vanillin catalyzed by UGT109A1, utilizing UDP-Glc as the glycosyl donor to furnish the glycosylated vanillin derivative; (**C**) LC-MS/MS characterization of the glycosylated product formed via UGT109A1-catalyzed glycosylation of vanillin; (**D**) Structure identification of the glycosylated product via NMR spectroscopy.

In this study, we successfully expressed the recombinant plasmid pET28a-UGT109A1 in *Escherichia coli* BL21 (DE3) and conducted an enzymatic activity assay to investigate glucovanillin biosynthesis. Our results showed that when vanillin was used as the aglycone substrate and UDP-glucose (UDP-Glc) acted as the glycosyl donor, a product labeled as P1 was formed through the catalytic activity of the recombinant UGT109A1 enzyme (Figure 1A). Liquid chromatography–tandem mass spectrometry (LC-MS/MS) analysis identified P1 with a molecular weight of 337.0899, corresponding to a molecular formula of C_14_H_18_O_8_. To further clarify the structural details of P1, tandem MS/MS fragmentation analysis was performed on the parent ion with a mass-to-charge ratio (*m*/*z*) of 337.0899. The resulting MS/MS spectrum displayed a dominant fragment ion at *m*/*z* 153.0549, which was assigned to the [M+H-C_6_H_10_O_5_]^+^ species. This fragment ion is derived from the loss of a hexose moiety (C_6_H_10_O_5_, molecular weight 162) from the protonated parent molecule. Notably, this fragmentation profile was found to be consistent with the characteristic mass spectral pattern of authentic glucovanillin (Figure 1C). Based on HPLC peak area quantification, the conversion rate of vanillin to glucovanillin was 70.05%, and the yield of glucovanillin was 51.18%.

Then, the molecular structure was confirmed by ^1^H and ^13^C NMR spectroscopy (Figure 1D, Figures S1 and S2). The presence of a β-D-glucopyranosyl moiety was clearly evidenced by the characteristic anomeric proton signal at δH 5.05 (1H, d, J = 7.6 Hz), which correlated to the anomeric carbon at δC 100.3 in the ^13^C NMR spectrum, confirming the β-configuration of the glycosidic linkage. Additional signals corresponding to the glucopyranosyl ring protons were observed in the range δH 3.39–3.86, while the corresponding carbons resonated at δC 61.0–77.0, including the primary alcohol carbon at δC 61.0 (C-6). Other characteristic resonances belonged to the aglycone moiety. The singlet signal at δH 9.81 was assigned to the aldehyde hydrogen, which corresponded to the carbonyl carbon at δC 191.6. The methoxy group exhibited a proton signal at δH 3.89 and a carbon signal at δC 55.2. Aromatic hydrogens and carbons were observed at δH 7.29–7.50 and δC 110.3–152.1, respectively. All NMR spectroscopic data were in good accordance with the speculated structure, which verified that one β-D-glucopyranosyl group was successfully linked to the aglycone skeleton.

To characterize the catalytic efficiency of UGT109A1 toward vanillin, the initial reaction velocities were measured at varying vanillin concentrations (Figure S3). Kinetic analysis revealed that UGT109A1 exhibited a K_m_ value of 1.496 mM for vanillin, indicating a moderate substrate affinity. The V_max_ was determined to be 17.47 µM·min^−1^, with a turnover number kc_at_ of 0.59 s^−1^ and a catalytic efficiency (kc_at_/K_m_) of 0.396 s^−1^·mM^−1^. These results demonstrate that UGT109A1 is capable of catalyzing the glucosylation of vanillin with reasonable efficiency, supporting its potential for enzymatic synthesis of glucovanillin.

Having successfully synthesized and characterized glucovanillin, we next sought to systematically evaluate its potential anti-T2DM mechanisms. Given that the therapeutic effects of natural products are often mediated through multiple targets and pathways, we employed network pharmacology, machine learning, and immune infiltration analysis to identify the core targets and molecular mechanisms underlying glucovanillin’s anti-T2DM activity.

### 2.2. Network Pharmacology Analysis of the Potential Targets of Glucovanillin in Improving T2DM

**Figure 2:**
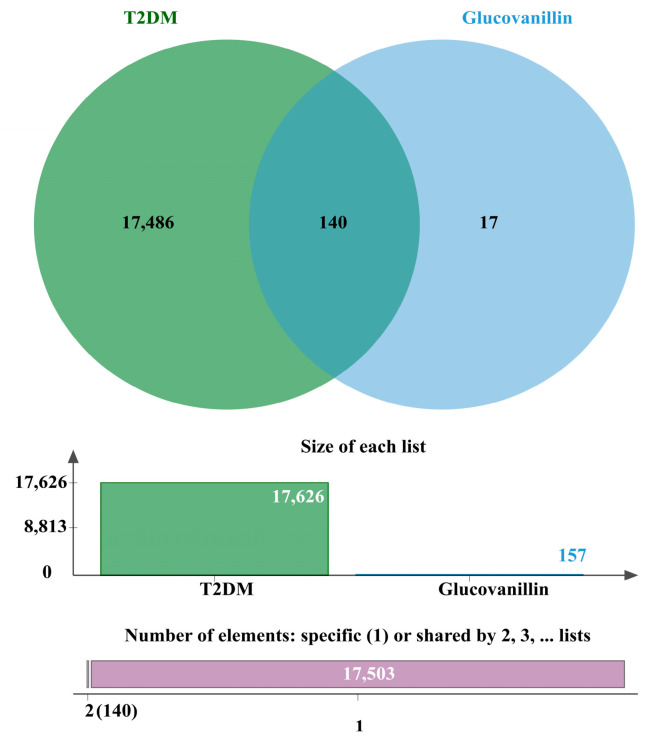
Venn diagram showing the overlap between glucovanillin-related targets and T2DM-related genes.

Network pharmacology, as a cutting-edge technology for systematically predicting drug targets and potential mechanisms of action, provides strong support for exploring the molecular mechanisms through which glucovanillin improves T2DM [25]. In this study, potential targets of glucovanillin were screened using three major databases—TargetNET, Swiss Target Prediction, and SEA—leading to the identification of 157 potential targets. Meanwhile, T2DM-related disease targets were gathered from the GeneCards and OMIM databases, resulting in a total of 17626 relevant targets after filtering duplicates. A Venn diagram was employed to analyze the overlap between these two target sets and visually display the findings (Figure 2). In the end, 140 shared targets were identified as potential key regulatory targets of glucovanillin in T2DM.

### 2.3. Functional Enrichment Analysis of Glucovanillin Targets in Improving T2DM

**Figure 3:**
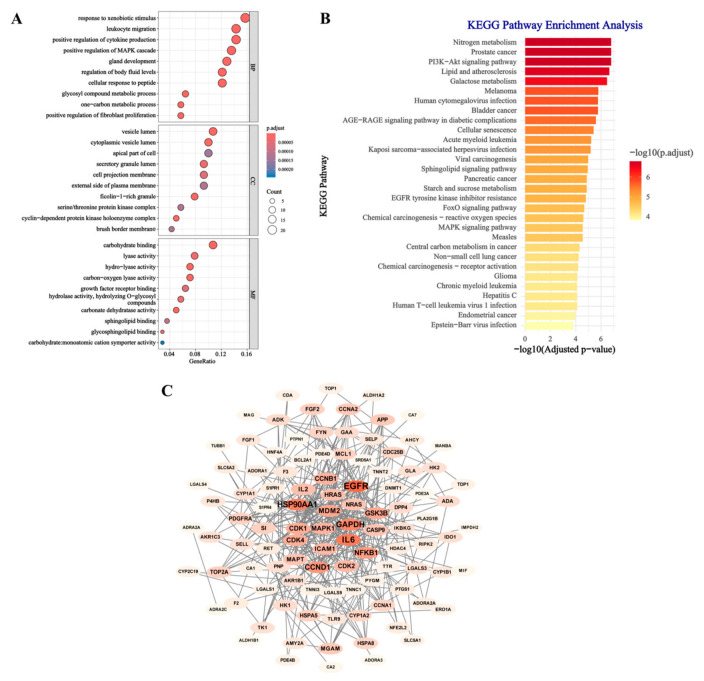
Gene Ontology (GO) and Kyoto Encyclopedia of Genes and Genomes (KEGG) pathway enrichment analysis of glucovanillin targets in improving T2DM. (**A**) Enrichment analysis results for Biological Process (BP), Cellular Component (CC), and Molecular Function (MF); (**B**) KEGG pathway enrichment analysis showing the main enriched pathways associated with the targets; (**C**) Protein–Protein Interaction (PPI) network of key targets.

To investigate how glucovanillin alleviates T2DM, enrichment analysis was performed on the 140 overlapping target genes identified from the previous screening to explore their biological significance. The top 10 Gene Ontology (GO) terms with the lowest false discovery rate (FDR) were selected for visualization (Figure 3A). In the Biological Process (BP) category, the target genes were notably enriched in pathways related to the response to xenobiotic stimuli and leukocyte migration. In the Cellular Component (CC) category, they were primarily enriched in the vesicle lumen and cytoplasmic vesicle lumen. Regarding Molecular Function (MF), significant enrichment was found in carbohydrate binding and lyase activity. KEGG pathway enrichment analysis highlighted that these genes were primarily involved in nitrogen metabolism and prostate cancer pathways (Figure 3B). A protein–protein interaction (PPI) network was constructed for the 140 candidate genes, consisting of 104 nodes and 301 edges. Key hub genes with high connectivity, such as HSP90AA1, EGF2, and IL6, were identified within the network (Figure 3C). The GO and KEGG enrichment analyses provide mechanistic insights into the potential anti-T2DM effects of glucovanillin. The enrichment of ‘response to xenobiotic stimuli’ in the BP category suggests that glucovanillin may modulate cellular detoxification or metabolic adaptation responses. Notably, the identification of ‘nitrogen metabolism’ as a key KEGG pathway is highly relevant, as dysregulated amino acid metabolism is intrinsically linked to insulin resistance and impaired glucose homeostasis in T2DM. While pathways such as ‘prostate cancer’ were also enriched, it is important to note that these pathways share critical signal transduction components, such as the PI3K/Akt and MAPK cascades, which are indispensable for insulin signaling and pancreatic β-cell function. Collectively, these findings suggest that glucovanillin may alleviate T2DM by exerting a multi-target, multi-pathway regulatory effect on metabolic homeostasis and insulin signaling networks.

### 2.4. Identification of Key Targets Based on Machine Learning

**Figure 4:**
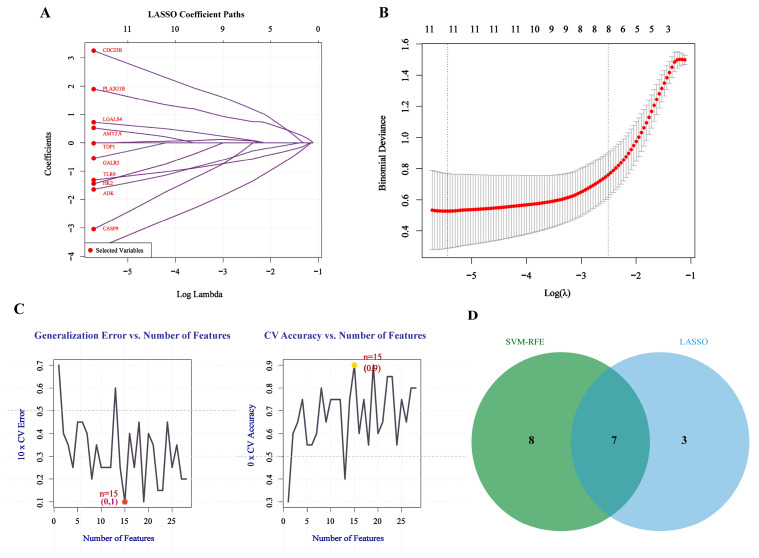
Identification of core target genes for glucovanillin in improving T2DM based on machine learning algorithms (**A**,**B**) LASSO logistic regression analysis. The *x*-axis represents the number of model genes corresponding to different λ values, with 8 genes identified at the minimum λ value; (**C**) Core genes were selected using the Support Vector Machine–Recursive Feature Elimination (SVM-RFE) algorithm, resulting in 15 candidate genes. The dashed horizontal lines indicate the reference threshold of 0.5 for CV error and CV accuracy, and the highlighted point marks the optimal feature number identified by the SVM-RFE model; (**D**) Venn diagram showing the intersection between the LASSO and SVM-RFE algorithms, identifying the seven common core targets of glucovanillin in improving T2DM.

With the aim of identifying core genes that mediate the T2DM-improving activity of glucovanillin, the 140 associated targets of this compound were subjected to analysis using two complementary machine learning algorithms: LASSO and SVM. The LASSO logistic regression model with triple cross-validation and penalization parameter adjustment was used to screen 8 potential core targets (Figure 4A,B). Subsequently, the SVM regression algorithm was employed to further filter and identify 15 core targets (Figure 4C). A Venn diagram was used to integrate the results from both methods, ultimately identifying seven key core targets for glucovanillin in improving T2DM: SLC5A1, CDC25B, PLA2G1B, TLR9, ADK, AMY2A, and LGALS4 (Figure 4D).

### 2.5. Expression Validation and Diagnostic Efficacy Evaluation of Seven Key Targets in T2DM

**Figure 5:**
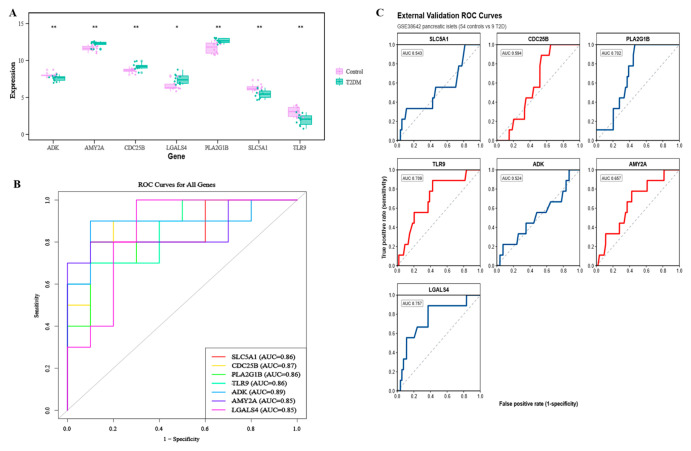
RNA sequencing analysis of core gene expression in T2DM patients and normal control samples. (**A**) Expression levels of the seven key targets in T2DM patient samples and healthy controls (GSE20966 dataset). * *p* < 0.05; ** *p* < 0.01; (**B**) Diagnostic efficacy of the seven core targets in distinguishing normal controls from T2DM samples, assessed using Receiver Operating Characteristic (ROC) curves. (**C**) External validation of the diagnostic efficacy of the seven core targets using an independent cohort (GSE38642 dataset).

To validate the expression levels of the core genes, RNA-seq data from T2DM patients in the GSE20966 dataset were retrieved from the publicly available GEO database. A comparison with normal samples revealed significant differences in the expression of SLC5A1, CDC25B, PLA2G1B, TLR9, ADK, AMY2A, and LGALS4 in the T2DM patient samples (Figure 5A). The diagnostic performance of these seven core genes was evaluated by constructing Receiver Operating Characteristic (ROC) curves and calculating the Area Under the Curve (AUC). The AUC values for SLC5A1, CDC25B, PLA2G1B, TLR9, ADK, AMY2A, and LGALS4 were 0.86, 0.87, 0.86, 0.86, 0.89, 0.85, and 0.85, respectively (Figure 5B). These findings underscore the significant diagnostic and pathophysiological relevance of these seven core genes for T2DM. To further validate the diagnostic efficacy and ensure the robustness of the identified core targets, an independent validation was performed using the GSE38642 dataset (54 controls vs. 9 T2D donors). The ROC analysis demonstrated that the 7 core genes maintained consistent diagnostic performance, effectively distinguishing T2DM patients from healthy controls (Figure 5C). This external validation confirms that our diagnostic model is reliable and not susceptible to overfitting.

This external validation confirms that our diagnostic model is reliable and not susceptible to overfitting. Given that several of these core genes are known to be involved in immune regulation, we next investigated the relationship between these seven core genes and immune cell infiltration in T2DM.

### 2.6. Correlation Analysis of Core Genes in T2DM with Immune Cell Infiltration

**Figure 6:**
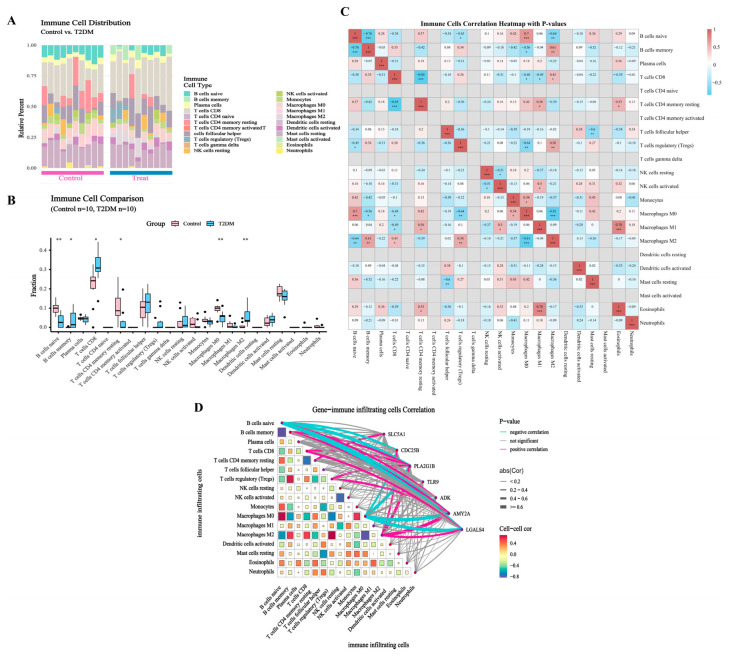
Immune Infiltration Analysis of T2DM Patients and Normal Controls Based on GSE20966 Dataset. (**A**,**B**) Single-sample Gene Set Enrichment Analysis (ssGSEA) of 22 immune cell types in the T2DM group and normal control group; (**C**) Correlation between various immune cell types in the T2DM group. * *p* < 0.05; ** *p* < 0.01; *** *p* < 0.001; (**D**) Correlation between seven core target genes and immune cell infiltration in the T2DM group. Correlation between the seven core target genes and immune cell infiltration in the T2DM group. The colors of the squares indicate the direction and magnitude of cell-cell correlations, and the square sizes represent the absolute correlation strength.

Differences in the composition of 22 immune cell types between normal control and T2DM patient samples, as well as their correlations with the seven core genes, were investigated using Single Sample Gene Set Enrichment Analysis (ssGSEA). The analysis revealed significant differences in the infiltration levels of native B cells, memory B cells, CD8 T cells, and M2 macrophages (Figure 6A,B). Additionally, specific correlations were observed between immune cell types (Figure 6C), such as a positive correlation between native B cells and M0 macrophages, and a negative correlation between resting mast cells and follicular helper T cells. Further analysis of the relationship between the seven core targets and immune cell infiltration (Figure 6D) showed that LGALS4 and AMY2A were positively correlated with M2 macrophages, suggesting that these two genes may participate in the development of insulin resistance or the regulation of pancreatic β-cell function in T2DM by modulating M2 macrophage polarization or function. CDC25B and PLA2G1B exhibited a unique immune modulation pattern, showing a negative correlation with activated B cells, but a strong negative correlation with M2 macrophages. This suggests that these two genes may exert a specific negative regulatory role in the T2DM immune microenvironment by bidirectionally inhibiting adaptive immunity, particularly different branches of B cells and M2 macrophages.

Given the established correlations between these core genes and T2DM pathology, we next sought to validate whether glucovanillin could directly bind to these key targets. Molecular docking was therefore performed to predict the binding affinities and interaction patterns between glucovanillin and the seven core proteins.

### 2.7. Molecular Docking of Glucovanillin with Core Targets

Molecular docking is a key technique in computer-aided drug design, which depends on the availability of known structures for both ligands and target proteins [26,27]. This method allows for the placement of small molecules within the three-dimensional framework of a target protein. After optimizing the positioning of the receptor–ligand complex, the conformation that most favorably facilitates interaction between the small molecule and the target macromolecule is chosen [28,29]. To computationally validate the predicted interactions between glucovanillin and the seven core targets identified through network pharmacology and machine learning, molecular docking analysis was conducted.

**Figure 7:**
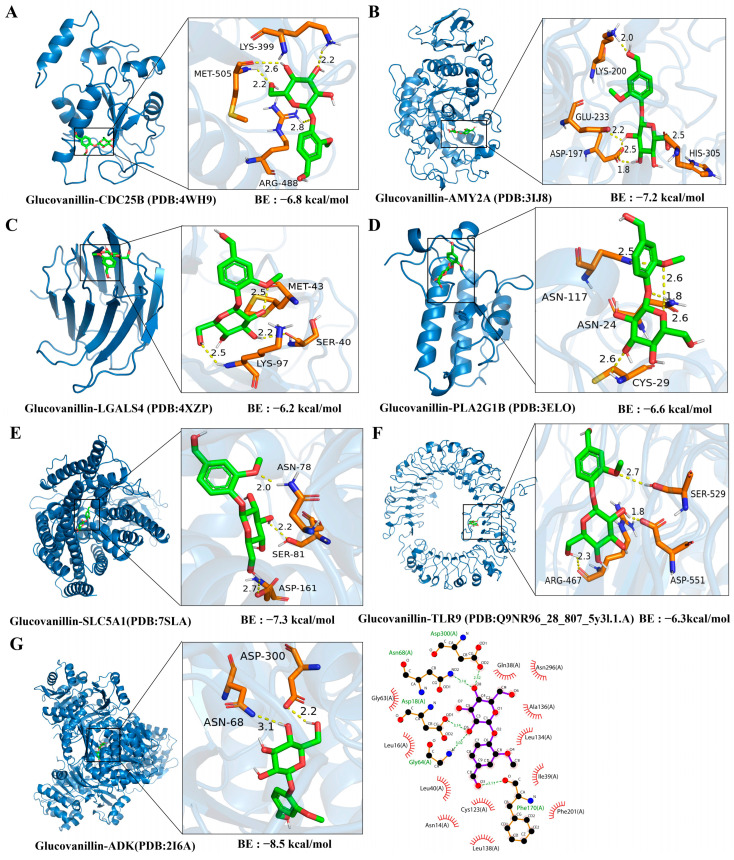
Molecular docking between glucovanillin and key targets. (**A**) Glucovanillin–CDC25B; (**B**) Glucovanillin–AMY2A; (**C**) Glucovanillin–LGALS4; (**D**) Glucovanillin–PLA2G1B; (**E**) Glucovanillin–SLC5A1; (**F**) Glucovanillin–TLR9; (**G**) Glucovanillin–ADK.

According to the docking results, glucovanillin is capable of interacting with the binding sites of various target proteins, demonstrating a strong affinity for all key targets (Figure 7). The molecular docking energies against different targets range from −6.2 to −8.5 kcal/mol, consistent with the principle that lower docking energies indicate stronger binding between glucovanillin and the respective targets. Among these interactions, the glucovanillin–ADK (PDB ID: 2I6A) complex exhibited the strongest binding affinity, with an energy value of −8.5 kcal/mol, indicating the most stable complex among all tested systems. This stability is reinforced by hydrogen bonds formed between glucovanillin and the ASN-68 and ASP-300 residues of the ADK receptor, as well as hydrophobic interactions with GLY-63, LEU-16, LEU-40, ASN-14, LEU-138, CYS-123, and PHE-201 residues of ADK. Therefore, it is hypothesized that glucovanillin may exert its hypoglycemic effect by modulating the ADK target.

### 2.8. Single-Cell Transcriptomic Landscape and Intercellular Communication Network of Core Targets in PBMCs

**Figure 8:**
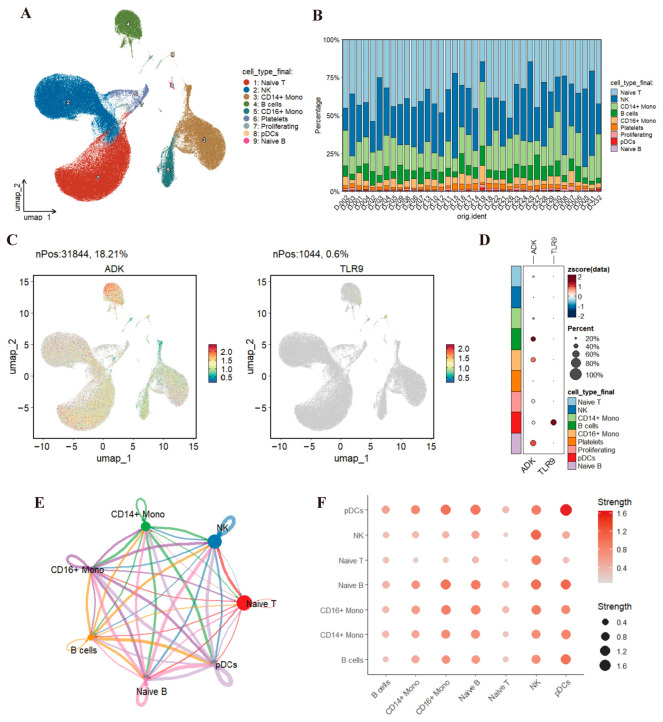
Single-cell expression landscape and intercellular communication network of core targets. (**A**) UMAP visualization of integrated immune cell clusters; (**B**) Proportional distribution of cell types across 10 sample batches; (**C**) Feature plots of ADK and TLR9 expression levels within the UMAP embedding; (**D**) Dot plot of average expression and percentage of ADK and TLR9 across annotated cell clusters; (**E**) Global network visualization of cell–cell communication between immune subsets; (**F**) Dot plot of specific interaction strengths between pairwise cell populations inferred by CellChat.

Unsupervised clustering of PBMC single-cell transcriptomes resolved major immune and blood-associated populations, including Naive T cells, NK cells, CD14^+^ monocytes, CD16^+^ monocytes, B cells, Naive B cells, pDCs, platelets, and a proliferating cluster (Figure 8A). The proportion of these cell types remained relatively stable across different samples, as shown in the bar plot (Figure 8B), indicating a consistent immune landscape that supports the reliability of downstream expression analyses. To further delineate the metabolic and immunological profiles of these populations, we examined the expression of ADK and TLR9. Consistent with the feature-level visualization (Figure 8C), dot plot analysis (Figure 8D) demonstrated that ADK exhibited a broad expression profile across most immune subsets, with significantly higher transcript levels specifically enriched in B cells and Naive B cells, as evidenced by both high expression percentage and average intensity within the B-lineage compartment. This distribution pattern suggests a robust adenosine metabolism program operating within these cells, potentially reflecting the high energetic demands and specialized signaling requirements of B cell activation and antibody production. In contrast, TLR9 expression was highly restricted, with a prominent and specific signal observed exclusively in pDCs (Figure 8C,D), where the quantitative metrics confirmed both high detection percentage and expression intensity solely within this cluster. This strict compartmentalization underscores TLR9’s specialized role as a sensor for nucleic acids within the innate immune surveillance system, enabling pDCs to detect viral or endogenous DNA and initiate rapid type I interferon responses.

To elucidate the coordination of systemic immune responses beyond individual cell-type signatures, intercellular communication inference was performed using curated ligand–receptor interactions. A dense and complex signaling interactome was revealed among the identified clusters (Figure 8E), with specific attention directed toward the communication axis between pDCs and B-lineage compartments. In the interaction heatmap (Figure 8F), pronounced communication was identified between these two populations, positioning B cells and Naive B cells as primary recipients of pDC-derived signals. Given that TLR9 expression is restricted to pDCs, it is postulated that nucleic-acid sensing is translated by pDCs into systemic signals that specifically target the lymphoid arm, establishing the pDC–B cell axis as a critical junction for integrating innate sensing with adaptive modulation. This concentrated crosstalk appears mechanistically underpinned by a distinct metabolic gene program; the broad and elevated expression of ADK within B-lineage clusters is consistent with their role as hubs for intercellular crosstalk, where adenosine metabolism may regulate signaling intensity and activation thresholds. Consequently, ADK is positioned as a fundamental metabolic regulator that facilitates the signaling receptivity and functional connectivity of the B-cell lineage, thereby governing immune coordination within the circulating pool.

These single-cell data provide cell-type-resolved support for the relevance of ADK in immune regulation. Although ADK transcripts were detectable across multiple immune subsets, their specific enrichment in B cells and Naive B cells indicates a strengthened adenosine metabolism program within the B-lineage compartment that couples metabolic state with higher signaling receptivity. Notably, the communication inference suggests that these metabolically primed B-lineage populations act as prominent recipients within the inferred immune interactome, reinforcing ADK as a functionally meaningful metabolic regulator in the circulating immune landscape and supporting its prioritization as an important therapeutic target for modulating immune–metabolic interactions in disease contexts.

### 2.9. Inhibitory Activity and Mechanism of Glucovanillin Against α-Glucosidase

**Figure 9:**
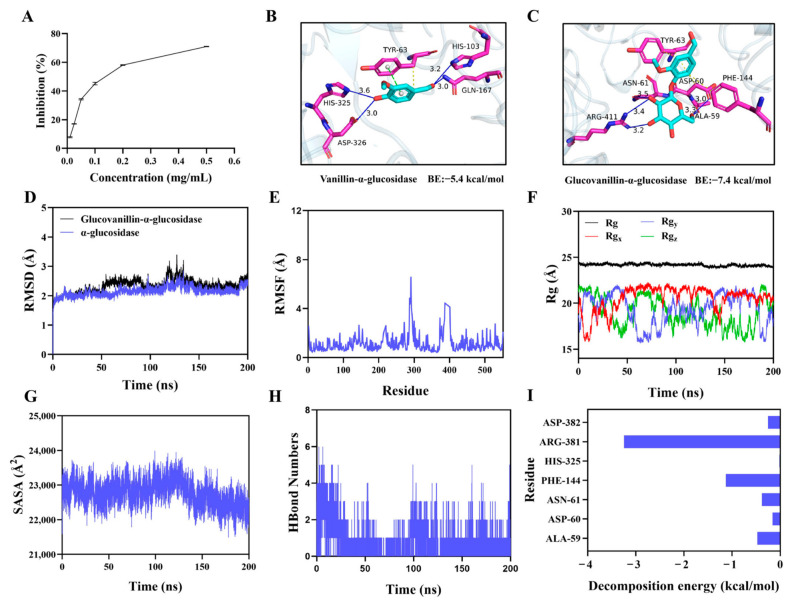
Molecular docking, molecular dynamics simulation, and MM/PBSA of vanillin and glucovanillin with *α*-glucosidase. (**A**) Concentration-dependent inhibition curve of *α*-glucosidase by glucovanillin; (**B**) Molecular docking of vanillin with *α*-glucosidase; (**C**) molecular docking of glucovanillin with *α*-glucosidase; (**D**) RMSD of the complex glucovanillin/*α*-glucosidase; (**E**) RMSF of the complex glucovanillin/*α*-glucosidase; (**F**) Rg of the complex glucovanillin/*α*-glucosidase; (**G**) SASA of the complex glucovanillin/*α*-glucosidase; (**H**) Hydrogen bond numbers of the complex glucovanillin/*α*-glucosidase; (**I**) Decomposition of binding free energy of residues for *α*-glucosidase.

In addition to the seven core targets identified above, *α*-glucosidase is a well-established therapeutic target for postprandial blood glucose control in T2DM. Given that vanillin, the aglycone precursor of glucovanillin, has been reported to inhibit *α*-glucosidase [11], we next investigated whether glycosylation enhances this inhibitory activity. The results showed that glucovanillin exhibited inhibitory activity against *α*-glucosidase in a concentration-dependent manner (Figure 9A). As the concentration increased from 0 mg/mL to 0.5 mg/mL, the inhibition rate gradually increased from approximately 7% to 71%. Its IC_50_ value was 413.84 ± 12.80 μM, suggesting moderate inhibitory potential.

Molecular docking serves as a valuable technique in computer-aided drug research, allowing researchers to assess the binding interactions between drug candidates and their potential molecular targets. This method provides essential insights into the possible therapeutic effectiveness of drugs in treating specific diseases. In this study, we utilized molecular docking to explore the interaction of glucovanillin with *α*-glucosidase, aiming to further investigate and confirm its hypoglycemic potential. Additionally, to determine whether glucovanillin offers a more effective inhibitory action on *α*-glucosidase, we employed vanillin, an unmodified *α*-glucosidase inhibitor, as a positive control. A comparative experiment was carried out to evaluate the binding profiles and functional differences between the two compounds. The molecular docking results indicate that the vanillin–*α*-glucosidase complex (Figure 9B) has a binding energy of −5.4 kcal/mol. The interaction is mainly driven by hydrogen bonds with residues like HIS-325, ASP-326, HIS-103, and GLN-167, along with a salt bridge and hydrophobic interactions involving TYR-63. This binding pattern mirrors that of natural phenolic inhibitors [30]. In contrast, the glucovanillin–*α*-glucosidase complex (Figure 9C) demonstrates a binding energy of −7.4 kcal/mol, reflecting a notably stronger binding affinity. Along with hydrophobic interactions involving TYR-63 and ASP-60, the binding is further stabilized by several hydrogen bonds with residues such as ARG-411, ASN-61, ASP-60, ALA-59, and PHE-144. As a result, glucovanillin forms a more stable complex with *α*-glucosidase, benefiting from a more extensive network of residue interactions and exhibiting a substantially lower binding energy compared to vanillin. This suggests that glucovanillin could potentially act as a more effective inhibitor of *α*-glucosidase.

To further assess the binding stability and MD characteristics of glucovanillin with *α*-glucosidase, a 200 ns MD simulation was performed. The time-dependent root mean square deviation (RMSD) (Figure 9D) shows that the conformational fluctuations of the glucovanillin–*α*-glucosidase complex are generally greater than those of the apo *α*-glucosidase; however, both systems remain stable within the 1.5–3.0 Å range. This suggests that upon complex formation, the enzyme undergoes adaptive conformational changes while maintaining its overall structural integrity. The root mean square fluctuation (RMSF) (Figure 9E) reveals localized flexibility fluctuations in certain residues near the binding site, which are linked to the conformational rearrangements of the active pocket triggered by ligand binding. These local flexibility changes are crucial for the functional inhibition of the target by the inhibitor. The radius of gyration (Rg) (Figure 9F) data indicates that the Rg value of the complex remains steady between 2.0 and 2.1 nm, demonstrating good structural compactness without significant unfolding. This suggests that glucovanillin binding does not disrupt the core structure of the enzyme. The dynamic changes in the solvent-accessible surface area (SASA) (Figure 9G) show that the solvent exposure on the surface of the complex remains stable, implying that the hydrophilic–hydrophobic interface of the enzyme is not significantly altered after ligand binding, which further supports the stability of the complex. The hydrogen bond (HBond) data (Figure 9H) show that the number of hydrogen bonds in the complex remains stable between 3 and 5 throughout the simulation, providing key intermolecular forces that stabilize the complex.

To quantitatively evaluate the binding affinity, the MM/PBSA method was used in this study to calculate the binding free energy, which is widely recognized as an effective approach for predicting the strength of protein–ligand interactions. The binding free energy and residue decomposition analysis (Table 1 and Figure 9I) revealed that the total binding free energy (ΔG_total_) of the glucovanillin–*α*-glucosidase complex is −10.17 ± 1.19 kcal/mol. Further residue decomposition analysis identified residues like ARG-381 and PHE-144 as the primary contributors to the binding free energy. Notably, the positively charged side chain of ARG-381 forms strong polar interactions with the hydroxyl group of glucovanillin, while the aromatic ring of PHE-144 stabilizes the ligand’s conformation through hydrophobic stacking. This “polar interaction + hydrophobic stacking” synergistic effect closely resembles the key binding mechanism observed in *α*-amylase inhibitors as reported by Rasouli et al. [30].

In conclusion, glucovanillin, through glucosyl modification, constructs a more enriched network of residue interactions, and the complex formed with *α*-glucosidase shows significantly superior binding stability and affinity compared to vanillin. The excellent dynamic stability demonstrated by MD simulations and MM/PBSA not only provide a solid molecular basis for understanding the *α*-glucosidase inhibitory activity of glucovanillin, but also offer important references for the development of efficient hypoglycemic candidate molecules based on structural modifications of NPs.

### 2.10. Analysis of Pharmacokinetic Prediction

ADMET analysis is a crucial tool in drug discovery, providing a comprehensive assessment of the pharmacokinetic and safety profiles of compounds [31,32]. In this study, we utilized the ADMET-AI platform to conduct in silico evaluation of the ADMET properties of the compound glucovanillin. It should be noted that all obtained results are purely computational predictions and require further experimental verification. The results reveal that glucovanillin exhibits favorable pharmacokinetic characteristics (Table 2). Specifically, its human intestinal absorption value is 0.54, indicating good permeability in the intestines; its oral bioavailability is 0.68, suggesting effective systemic exposure after oral administration. The plasma protein binding rate is 43.39%, reflecting a balanced distribution between the free drug and a “reservoir” for sustained release. The blood–brain barrier penetration value is 0.48, which implies a possible tendency to cross the blood–brain barrier. This property may be relevant to the treatment of diabetic-related neuropathy, though further validation is needed. Furthermore, glucovanillin demonstrates excellent biocompatibility, with very low risks for clinical toxicity (0.07), mutagenicity (0.37), and carcinogenicity (0.03). It also shows minimal risks for hERG-mediated cardiac toxicity (0.10) and drug-induced liver injury (0.33). These findings suggest that glucovanillin could be a promising candidate for blood glucose-lowering therapies.

### 2.11. Limitations and Future Perspectives

Although this study systematically revealed the anti-T2DM mechanism of glucovanillin, several limitations still need to be addressed. First, the α-glucosidase inhibitory activity of glucovanillin is moderate, with a high micromolar IC_50_ value (413.84 ± 12.80 μM). Such a high working concentration limits its direct application as a high-potency clinical drug candidate at the current stage, and high micromolar concentrations theoretically carry a potential risk of non-specific interference and off-target effects. However, multiple lines of evidence including molecular docking, MD simulation, MM/PBSA and residue decomposition analysis verification proved that the inhibitory effect of glucovanillin is based on specific molecular recognition rather than universal disruption of the assay system.

Second, all computational predictions and in vitro enzyme assays in this work have not been further verified by in vivo animal experiments, and the actual effective concentration, tissue distribution and long-term toxicity of glucovanillin in vivo remain unclear. Combined with the ADMET prediction results that glucovanillin has good oral bioavailability and low toxicity, we propose three directions for follow-up research: (1) Further structural modification of glucovanillin to reduce its effective inhibitory concentration and improve potency; (2) carry out in vivo T2DM animal model experiments to evaluate the hypoglycemic effect, effective dose and off-target risk of glucovanillin in living organisms; (3) explore the application potential of glucovanillin as a lead compound or adjuvant hypoglycemic component in functional foods and health products.

## 3. Method

### 3.1. Heterologous Expression and Purification of UGT109A1

The gene encoding UGT109A1 was synthesized by GENEWIZ and subsequently cloned into the pET28a(+) expression vector, to generate the recombinant plasmid pET28a-UGT109A1. This plasmid was then introduced into *Escherichia coli* BL21 (DE3) competent cells, where the target protein was expressed. The expression conditions for glycosyltransferase UGT109A1 were optimized as follows: the cells were cultured in LB medium containing 50 mg/L kanamycin at 37 °C for approximately 4 h, or until the optical density at 600 nm (OD_600_) reached between 0.6 and 0.8. Subsequently, isopropyl-β-D-thiogalactopyranoside (IPTG) was added to a final concentration of 0.5 mM, and the culture was incubated at 18 °C with shaking at 180 rpm for 16 h to induce protein expression. After induction, the cells were harvested by centrifugation, resuspended in 10 mM imidazole aqueous solution, and subjected to sonication for cell lysis. The resulting supernatant containing the target protein was applied to a Ni-NTA affinity chromatography column for purification The protein was eluted from the column using a linear gradient of imidazole, ranging from 40 to 400 mM.

### 3.2. Enzyme Activity Assay

A 100 μL reaction mixture was prepared containing 0.5 mM vanillin, 10 μg of glycosyltransferase UGT109A1, 5 mM UDP-Glc, and 100 mM phosphate buffer (pH 7.5), and incubated at 37 °C for 12 h. An equal volume of chromatographic methanol was added to terminate the reaction. The mixture was centrifuged at 12,000 rpm for 15 min, and the resulting supernatant was transferred to a sample vial for subsequent analysis.

The glycosylation products were identified by HPLC and LC-MS/MS. The analytical system consisted of an Agilent 1290 ultra-high-performance liquid chromatograph (UPLC) coupled with an Agilent 6545 quadrupole time-of-flight mass spectrometer (Q-TOF-MS) equipped with an electrospray ionization (ESI) source (Agilent Technologies, Santa Clara, CA, USA). A C18 column was used, with an injection volume of 10 μL. The mobile phase flow rate was 1 mL/min, and the column temperature was maintained at 35 °C. UV detection was used for HPLC analysis.

The retention times of vanillin and the product glucovanillin were 15.3 and 11.5 min, respectively. For kinetic parameter determination, reactions were performed with vanillin at concentrations ranging from 0.25 to 2.5 mM. Each 100 μL reaction system contained 5 mM UDP-glucose, 3 μg of purified UGT109A1 and 1 × PBS buffer, and incubated at 37 °C. Reactions were stopped by adding 100 μL of methanol. Kinetic data were fitted to the Michaelis–Menten equation by nonlinear regression using GraphPad Prism 11.

### 3.3. Collection of Target Proteins for Glucovanillin

First, the chemical structure information and the Simplified Molecular Input Line Entry System (SMILES) string of glucovanillin were retrieved from the PubChem database (https://pubchem.ncbi.nlm.nih.gov/, accessed on 15 June 2026). Then, using Homo sapiens (human) as the species restriction, the potential targets of this compound were screened through three databases: TargetNET (http://targetnet.scbdd.com/, accessed on 15 June 2026), Swiss Target Prediction (http://swisstargetprediction.ch/, accessed on 15 June 2026), and SEA (https://sea.bkslab.org/, accessed on 15 June 2026). The targets obtained from the three databases were merged, duplicate entries were removed, and a comprehensive dataset of glucovanillin-related targets was finally constructed.

### 3.4. Screening of Disease Targets Associated with T2DM

The disease targets associated with T2DM were retrieved from two major databases: Online Mendelian Inheritance in Man (OMIM, https://omim.org/, accessed on 15 June 2026) and GeneCards (https://www.genecards.org/, accessed on 15 June 2026). During the retrieval process, “Type 2 Diabetes Mellitus” and “T2DM” were used as key terms, and the species was strictly limited to Homo sapiens. To eliminate false-positive background noise and ensure the high reliability of the disease-target network, a strict filtering criterion was applied: only targets with a relevance Score > 0.4 in the GeneCards database were retained. The valid search results from both databases were then combined, and duplicate target entries were excluded. As a result, a final comprehensive set of potential T2DM-related disease targets was obtained.

### 3.5. Functional Pathway Analysis of Target Genes

To elucidate the potential molecular mechanisms of glucovanillin in the treatment of T2DM, this study utilized the DAVID database (https://david.ncifcrf.gov/, accessed on 15 June 2026) to perform Gene Ontology (GO) functional annotation and KEGG pathway enrichment analysis on the core target genes identified in the screening process. DAVID, as a classic integrated bioinformatics analysis platform, consolidates multi-source biological data and specialized analytical tools, providing systematic and comprehensive functional annotation for large-scale gene or protein sets, thereby aiding researchers in uncovering potential biological significance. The analysis covered the three core dimensions of the GO database (biological process, cellular component, and molecular function) and KEGG signaling pathways. Significantly enriched functional terms and pathways were determined using a corrected *p*-value < 0.05 as the selection criterion.

### 3.6. Protein–Protein Interaction (PPI) Network Analysis

The protein–protein interaction (PPI) network was constructed using the STRING database (https://string-db.org/, accessed on 15 June 2026), to identifying potential key targets of glucovanillin in T2DM. To enhance the accuracy of the interaction data, a minimum confidence threshold of 0.7 was applied. The resulting raw PPI network data from STRING were then imported into Cytoscape 3.7.2 for visualization and analysis of the network topological structure. Modular analysis of the core PPI network was conducted using the MCODE plugin in Cytoscape, with the goal of identifying functionally related modules characterized by high clustering coefficients.

### 3.7. Core Target Screening Using Machine Learning Algorithms

To identify the key targets of glucovanillin in the treatment of T2DM, this study employed two machine learning techniques—least absolute shrinkage and selection operator (LASSO) regression and support vector machine (SVM). LASSO regression, a method that combines variable selection and regularization, was used to mitigate overfitting and improve the accuracy of the model predictions. SVM, a well-established supervised learning technique, was particularly useful for feature selection in classification and regression problems. To enhance the feature selection process, recursive feature elimination (RFE) was incorporated, which progressively eliminated less relevant variables to identify the optimal set for improving model performance. The core targets were identified as the genes selected by both algorithms. LASSO regression was performed using the ‘glmnet’ package in R (version 4.1-10) with the following settings: standardize = TRUE, alpha = 1, family = “binomial” (appropriate for binary classification of T2DM vs. control), and 3-fold cross-validation. The optimal penalty parameter (λ) was determined using the lambda.min criterion to minimize cross-validation error. For the SVM-RFE analysis, the ‘caret’ (version 6.0-94) and ‘e1071’ (version 1.7-14) packages were employed. A linear kernel was selected to ensure the linearity of feature separation, and the model was trained using a 10-fold cross-validation strategy with three repeats to ensure the stability of feature selection. This combined approach effectively minimized false positives, ensuring that the identified biomarkers possess strong discriminative power and robustness.

### 3.8. Differential Expression and Diagnostic Efficacy Evaluation of Core Genes

In this study, the GSE20966 dataset, which includes transcriptomic data from 10 non-diabetic and 10 T2DM human pancreatic β-cell samples, was obtained from the GEO database for the analysis and visualization of core gene expression patterns. To eliminate systematic variations and ensure data consistency, the raw gene expression matrix underwent log2 transformation and quantile normalization using the ‘limma’ (version 3.62.0) R package prior to analysis. Core genes with significant differential expression between the groups were identified using a *p*-value threshold of <0.05. Additionally, mRNA expression data from both the control and T2DM groups were used to construct a Receiver Operating Characteristic (ROC) curves to assess the diagnostic potential of the selected core genes. The diagnostic accuracy was determined by calculating the area under the curve (AUC). For model optimization and to prevent overfitting, a 3-fold cross-validation strategy was implemented during the machine learning feature selection process. The diagnostic performance was rigorously assessed in both the discovery cohort (GSE20966, *n* = 20) and the independent external validation cohort (GSE38642, *n* = 63), ensuring the robustness and generalizability of the identified biomarkers.

### 3.9. Immune Infiltration Analysis

To assess the differences in immune cell infiltration between T2DM patients and healthy controls, single-sample gene set enrichment analysis (ssGSEA) was conducted using the GSVA package (version 1.46.0) in R. Statistical differences in infiltration levels between groups were assessed using the Wilcoxon rank-sum test. Additionally, the correlation between the expression levels of core target genes and the infiltration levels of various immune cell types was further analyzed.

### 3.10. Single-Cell Analysis

Public PBMC single-cell RNA-seq data were processed in R using Seurat (v5-compatible workflow). The dataset used in this study was obtained from the Gene Expression Omnibus database under the accession number GSE268210, encompassing 10 distinct sequencing batches. Raw count matrices were imported and merged by sample (orig.ident). Initial quality control was performed to ensure the inclusion of only high-quality cells, after which only cells with more than 200 detected genes (nFeature_RNA > 200) and a mitochondrial content of less than 5% (percent.mt < 5%) were retained. Following quality control, raw count matrices were normalized using the NormalizeData function with the LogNormalize method and a scale factor of 10,000. To eliminate potential technical noise and batch effects across the 10 distinct batches, the ‘Harmony’ algorithm was employed for data integration, ensuring consistent cell-type clustering and biological signal preservation. To eliminate potential artifacts arising from heterotypic doublets, the DoubletFinder (v2.0) package was employed. Based on the experimental design and cell loading density, the expected doublet rate was set to 0.8%. Only cells classified as “singlets” were preserved, whereas predicted doublets were excluded from the dataset prior to downstream integration and clustering. Two-dimensional embeddings (UMAP/tSNE) were generated for visualization. Major immune cell types were annotated using canonical marker genes, and cell-type composition was summarized per donor or sample. Gene expression of ADK and TLR9 was visualized at single-cell resolution and compared across annotated cell types. Intercellular communication inference was performed with CellChat using curated ligand–receptor interactions; overexpressed ligands/receptors and overexpressed interactions were identified, communication probabilities were computed, and network visualizations were generated after excluding non-immune populations when appropriate to stabilize network topology and improve interpretability.

### 3.11. Molecular Docking, Molecular Dynamics (MD) Simulation, and MM/PBSA of Glucovanillin with α-Glucosidase

The 3D structure of glucovanillin was retrieved from the PubChem database (https://pubchem.ncbi.nlm.nih.gov/, accessed on 15 June 2026) and energy-minimized using Chem3D 23.1.1 software with the MM2 force field. The crystal structures of the target proteins were downloaded from the RCSB PDB database with the following accession IDs: ADK (2I6A), AMY2A (3IJ8), CDC25B (4WH9), LGALS4 (4XZP), PLA2G1B (3ELO), SLC5A1 (7SLA), and TLR9 (8AR3). Each protein structure was processed using PyMOL 2.6 to remove water molecules and phosphate groups. AutoDockTools 1.5.6 was then used to add polar hydrogens, assign Gasteiger charges, merge non-polar hydrogens, and define the docking grid boxes. Considering the distinct geometry of binding pockets, customized grid sizes were adopted for different targets: ADK possesses a compact and well-defined binding cavity, so its grid box was centered at (14.5, −19.011, 19.137) with dimensions of 15 × 15 × 15 Å^3^ to avoid false-positive poses. In contrast, AMY2A, CDC25B, LGALS4, PLA2G1B, SLC5A1 and TLR9 have large, open or extended binding clefts, thus larger grids were applied. For AMY2A, the grid box was centered at (8.449, 28.393, 49.294) with dimensions of 47.25 × 47.25 × 47.25 Å^3^. For CDC25B, the grid box was centered at (5.669, −11.259, −10.281) with dimensions of 47.25 × 47.25 × 47.25 Å^3^. For LGALS4, the grid box was centered at (80.378, 25.807, 3.266) with dimensions of 47.25 × 47.25 × 46.5 Å^3^. For PLA2G1B, the grid box was centered at (−13.124, −24.389, −0.652) with dimensions of 47.25 × 47.25 × 42.75 Å^3^. For SLC5A1, the grid box was centered at (124.599, 129.366, 124.967) with dimensions of 47.25 × 47.25 × 47.25 Å^3^. For TLR9, the grid box was centered at (17.178, 4.842, −24.141) with dimensions of 47.25 × 47.25 × 47.25 Å^3^. Molecular docking was performed using AutoDock Vina software with an exhaustiveness parameter of 32 and 9 independent docking runs per ligand–protein pair. The conformation with the lowest binding energy was selected for further analysis. The docking results were visualized using LigPlus+ v.2.3 and PyMOL 2.6 to generate 2D and 3D interaction diagrams between glucovanillin and key residues of each target protein.

In this study, MD simulation was carried out using GROMACS 2022. The ligand topology files were generated using the GAFF2 force field via sobtop_1.0(dev3.1) software, and electrostatic charges were assigned to the ligand using the RESP method. The receptor protein was parameterized with the AMBER14SB force field. For solvation, the system was modeled with the TIP3P water model, and a cubic water box with dimensions of 1 nm was utilized. Sodium (Na^+^) and chloride (Cl^−^) ions were added to the system to achieve a concentration of 0.15 M NaCl using the GROMACS gmx genion tool. Electrostatic interactions were calculated using the Particle Mesh Ewald (PME) method, with a cutoff distance of 1 nm. The force field and PME parameters were adjusted in accordance with GROMACS standards. Bond constraints were applied using the LINCS algorithm. Prior to the MD simulation, the system underwent an energy minimization process, which consisted of 3000 steps of steepest descent optimization followed by 2000 steps of conjugate gradient optimization. The simulation was performed at 310 K using the Nosé–Hoover thermostat, and pressure was maintained at 1 bar with the Parrinello–Rahman barostat. A 200 ns simulation was conducted in the NPT ensemble with an integration time step of 2 fs. Various GROMACS tools, including gmx rmsd, gmx rmsf, gmx hbond, gmx gyrate, and gmx sasa, were employed to compute metrics such as root-mean-square deviation (RMSD), root-mean-square fluctuation (RMSF), hydrogen bonds (H-bonds), radius of gyration (Rg), and solvent-accessible surface area (SASA). The binding free energy of the complex was calculated using the gmx_MMPBSA tool in GROMACS. The trajectory of the final 10 ns after the simulation reached equilibrium was used. A total of 201 frames were evenly extracted at intervals of 50 ps for binding free energy calculations.

### 3.12. Pharmacokinetic Prediction

The drug-like properties of glucovanillin were assessed using the ADMET-AI platform (https://admet.ai.greenstonebio.com/), accessed on 22 November 2025. The compound was uploaded into the ADMET-AI platform for analysis of its ADMET characteristics.

### 3.13. Statistical Analysis

All statistical analyses were performed using R software (v4.2.3) and GraphPad Prism 11 software. Quantitative data are expressed as the mean ± standard deviation (SD). For comparisons between two groups, the Wilcoxon rank-sum test was used. A *p*-value < 0.05 was considered statistically significant. For multiple comparisons in GO/KEGG enrichment and immune infiltration analysis, the Benjamini–Hochberg (BH) method was applied to control the false discovery rate (FDR), and a corrected *p*-value (FDR) < 0.05 was set as the threshold for significance.

## 4. Conclusions

This study successfully synthesized glucovanillin via enzymatic catalysis, establishing an eco-friendly and efficient strategy for its preparation. By integrating network pharmacology, machine learning and immune infiltration analysis, we identified seven core targets potentially associated with the therapeutic effects of glucovanillin against T2DM. These targets exhibited distinct expression patterns in T2DM patients, with AUC values ranging from 0.85 to 0.89, suggesting good diagnostic performance. Furthermore, the seven targets were closely correlated with the infiltration of immune cells including M2 macrophages. It is speculated that glucovanillin may regulate the immune microenvironment, thereby interfering with T2DM-related pathological processes such as insulin resistance and pancreatic β-cell dysfunction. Functional enrichment analysis showed that these targets participate in biological processes including response to exogenous stimuli and carbohydrate binding, as well as pathways such as nitrogen metabolism, which helps to elucidate the possible action mechanisms of glucovanillin.

In this study, we further verified the inhibitory effect of glucovanillin on *α*-glucosidase. The experimental results demonstrated that glucovanillin exhibits moderate concentration-dependent activity against *α*-glucosidase, with an IC_50_ of 413.84 ± 12.80 μM, confirming that glycosylation effectively improved the enzyme inhibitory ability of vanillin. Molecular docking analysis suggested that glucovanillin has a higher binding affinity to *α*-glucosidase (−7.4 kcal/mol) than its parent compound vanillin (−5.4 kcal/mol). Multiple hydrogen bonds and hydrophobic interactions were observed between glucovanillin and key residues (ARG-411, PHE-144), forming a stable interaction network that may contribute to its inhibitory effect on *α*-glucosidase. The stability of the glucovanillin–*α*-glucosidase complex was further assessed through 200 ns MD simulation and MM/PBSA calculations, with a calculated binding free energy of −10.17 ± 1.19 kcal/mol. ADMET prediction results indicated that glucovanillin possesses favorable pharmacokinetic properties, including good intestinal absorption, satisfactory oral bioavailability and moderate plasma protein binding. Meanwhile, the compound showed low predicted risks of toxicity, mutagenicity and carcinogenicity. Collectively, these in silico and in vitro results suggest that glucovanillin deserves further exploration as a promising lead compound for anti-T2DM research. Further structural optimization is required to reduce its effective concentration and mitigate potential off-target risks in follow-up studies.

In summary, this work verified that UGT109A1-catalyzed glycosylation of vanillin to produce glucovanillin can improve its bioactivity, target binding capacity and pharmacokinetic profile. We combined enzymatic synthesis, network pharmacology, machine learning, immune infiltration analysis, single-cell transcriptomics, in vitro enzyme assays, *α*-glucosidase inhibition activity assays, MD simulation and ADMET prediction to systematically explore the potential anti-T2DM mechanisms of glucovanillin at multiple levels. All computational findings in this study are preliminary predictions. Although the present results support the potential value of glucovanillin for T2DM treatment, further comprehensive in vivo experiments are still required for definitive validation. This work also provides a reference for structural modification of natural products in the research and development of drugs against metabolic diseases.

## Figures and Tables

**Table 1:** MM/PBSA based binding free energies of complex (kcal/mol).

Energy Components	Glucovanillin–*α*-Glucosidase
ΔG_vdw_	−25.68 ± 2.21
ΔG_ele_	−20.51 ± 3.84
ΔG_polar_	38.6 ± 3.01
ΔG_nonpolar_	−2.59 ± 0.10
ΔG_gas_	−46.18 ± 3.73
ΔG_solv_	36.01 ± 2.98
ΔG_total_	−10.17 ± 1.19

**Table 2:** ADMET profiling of compounds.

Compound	Glucovanillin
Human Intestinal Absorption	0.54
Oral Bioavailability	0.68
Aqueous Solubility	−1.27 log mol/L
Lipophilicity	−1.09 log-ratio
Hydration Free Energy	−17.57 kcal/mol
Cell Effective Permeability	−5.54 cm/s
PAMPA Permeability	0.19
P-glycoprotein Inhibition	0.01
Blood–Brain Barrier Penetration	0.48
Plasma Protein Binding Rate	43.39%
Volume of Distribution at Steady State	0/kg
CYP1A2 Inhibition	1.75 × 10^−3^
CYP2C19 Inhibition	0.02
CYP2C9 Inhibition	3.02 × 10^−3^
CYP2D6 Inhibition	3.17 × 10^−3^
CYP3A4 Substrate	0.62
CYP3A4 Inhibition	2.34 × 10^−4^
Half Life	11.70 h
hERG Blocking	0.10
Clinical Toxicity	0.07
Mutagenicity	0.37
Drug-Induced Liver Injury	0.33
Carcinogenicity	0.03
Acute Toxicity LD50	1.62 log(1/(mol/kg))
Skin Reaction	0.25
Androgen Receptor (Full Length)	0.03
Androgen Receptor (Ligand Binding Domain)	0.01
Aryl Hydrocarbon Receptor	2.70 × 10^−3^
Aromatase	0.01
Estrogen Receptor (Full Length)	0.13
Estrogen Receptor (Ligand Binding Domain)	0.01
Peroxisome Proliferator-Activated Receptor Gamma	1.25 × 10^−3^
Nuclear Factor (Erythroid-Derived 2)-Like 2/Antioxidant Responsive Element	0.02
ATPase Family AAA Domain-Containing Protein 5 (ATAD5)	2.06 × 10^−3^
Heat Shock Factor Response Element	7.23 × 10^−4^
Mitochondrial Membrane Potential	1.61 × 10^−3^
Tumor Protein p53	0.04

## Data Availability

Data is contained within the article or Supplementary Materials.

## Supplementary Materials

The following supporting information can be downloaded at: https://www.mdpi.com/article/10.3390/molecules31132228/s1, Figure S1: ^1^H NMR spectrum of P3 in methanol–d4 (600 MHz); Figure S2: ^13^C NMR spectrum of P3 in methanol–d4 (600 MHz); Figure S3: Enzyme kinetics of UGT109A1 toward vanillin.

## References

[1] Qi G.Y., Wang F., Shi Y.B., Feng J., Xu J. (2025). Analysis of postprandial time trends and influencing factors of blood glucose and insulin in type 2 diabetes mellitus (T2DM) with metabolic dysfunction-associated steatotic liver disease (MASLD): A retrospective study based on propensity score matching (PSM). Acta Diabetol..

[2] Cardullo N., Muccilli V., Pulvirenti L., Cornu A., Pouységu L., Deffieux D., Quideau S., Tringali C. (2020). C-glucosidic ellagitannins and galloylated glucoses as potential functional food ingredients with anti-diabetic properties: A study of *α*-glucosidase and *α*-amylase inhibition. Food Chem..

[3] Mattsson K., Pihlsgård M., Enhörning S., Timpka S. (2024). Incident Cardiovascular Disease in Women With Type 1 or Type 2 Diabetes Following a Hypertensive Disorder of Pregnancy. Hypertension.

[4] Shan X.X., Yao S.H., Hu B., Xu C., Cao Y.H., Dai W. (2025). A Prospective Study of the Correlation Between Time in Range and Incidence of Diabetic Cardiovascular Autonomic Neuropathy in Patients with Type 2 Diabetes Mellitus. Diabetes Metab. Syndr. Obes..

[5] Gange W.S., Lopez J., Xu B.N.Y., Lung K.A., Seabury S.A., Toy B.C. (2021). Incidence of Proliferative Diabetic Retinopathy and Other Neovascular Sequelae at 5 Years Following Diagnosis of Type 2 Diabetes. Diabetes Care.

[6] Ghasemi M., Iraji A., Dehghan M., Nosood Y.L., Ghanavieh N.F., Hashempur M.H., Mojtabavi S., Faramarzi M.A., Mahdavi M., Al-Harrasi A. (2025). Quinoline-piperazine derivatives as potential *α*-Glucosidase inhibitors: Synthesis, biological evaluation, and in silico studies. J. Mol. Struct..

[7] Takács I., Szekeres A., Takács A., Rakk D., Mézes M., Polyák A., Lakatos L., Gyémánt G., Csupor D., Kovács K.J. (2020). Wild Strawberry, Blackberry, and Blueberry Leaf Extracts Alleviate Starch-Induced Hyperglycemia in Prediabetic and Diabetic Mice. Planta Medica.

[8] Shah M.A., Khalil R., Ul-Haq Z., Panichayupakaranant P. (2017). *α*-Glucosidase inhibitory effect of rhinacanthins-rich extract from *Rhinacanthus nasutus* leaf and synergistic effect in combination with acarbose. J. Funct. Foods.

[9] Fei Z., Xu Y., Zhang G.Y., Liu Y., Li H., Chen L.X. (2024). Natural products with potential hypoglycemic activity in T2DM: 2019–2023. Phytochemistry.

[10] Sknepnek A., Miletic D., Stupar A., Salevic-Jelic A., Nedovic V., Kljakic A.C. (2025). Natural solutions for diabetes: The therapeutic potential of plants and mushrooms. Front. Nutr..

[11] Liu Y.J., Zhu J., Yu J.M., Chen X., Zhang S.Y., Cai Y.X., Li L. (2021). A new functionality study of vanillin as the inhibitor for *α*-glucosidase and its inhibition kinetic mechanism. Food Chem..

[12] Nanok K., Sansenya S. (2021). Combination effects of rice extract and five aromatic compounds against *α*-glucosidase, *α*-amylase and tyrosinase. J. Biosci. Bioeng..

[13] Zhang H.Y., Che X.C., Jing H.Y., Su Y.W., Yang W.Q., Wang R.B., Zhang G.Q., Meng J., Yuan W., Wang J. (2024). A New Potent Inhibitor against *α*-Glucosidase Based on an In Vitro Enzymatic Synthesis Approach. Molecules.

[14] Lu C., Zhao S.J., Wei G.N., Zhao H.J., Qu Q.L. (2017). Functional regulation of ginsenoside biosynthesis by RNA interferences of a UDP-glycosyltransferase gene in *Panax ginseng* and *Panax quinquefolius*. Plant Physiol. Biochem..

[15] He Q.L., Yin H., Jiang J.J., Bai Y.F., Chen N., Liu S.W., Zhuang Y.B., Liu T. (2017). Fermentative Production of Phenolic Glucosides by *Escherichia coli* with an Engineered Glucosyltransferase from *Rhodiola sachalinensis*. J. Agric. Food Chem..

[16] Yu H., Zhou J., Zhang J., He X.Y., Peng S.Q., Ling H., Dong Z., Lu X.Y., Tian Y., Guan G.P. (2024). Functional Identification of *HhUGT74AG11*—A Key Glycosyltransferase Involved in Biosynthesis of Oleanane-Type Saponins in *Hedera helix*. Int. J. Mol. Sci..

[17] Ren C.H., Guo Y., Xie L.F., Zhao Z.K., Xing M.Y., Cao Y.L., Liu Y.L., Lin J., Grierson D., Zhang B. (2022). Identification of UDP-rhamnosyltransferases and UDP-galactosyltransferase involved in flavonol glycosylation in *Morella rubra*. Hortic. Res..

[18] He B., Bai X., Tan Y.M., Xie W.T., Feng Y., Yang G.Y. (2022). Glycosyltransferases: Mining, engineering and applications in biosynthesis of glycosylated plant natural products. Synth. Syst. Biotechnol..

[19] Jiang Z.N., Chen N.H., Wang H.T., Tian Y.G., Du X.Y., Wu R.B., Huang L.Q., Wang Z.L., Yuan Y. (2025). Molecular characterization and structural basis of a promiscuous glycosyltransferase for β-(1,6) oligoglucoside chain glycosides biosynthesis. Plant Biotechnol. J..

[20] Liang H.C., Hu Z.F., Zhang T.T., Gong T., Chen J.J., Zhu P., Li Y., Yang J.L. (2017). Production of a bioactive unnatural ginsenoside by metabolically engineered yeasts based on a new UDP-glycosyltransferase from *Bacillus subtilis*. Metab. Eng..

[21] Zhou C., Chen T.J., Gu A.D., Hu Z.F., Li Y., Gong T., Chen J.J., Yang J.L., Zhu P. (2023). Combining protein and metabolic engineering to achieve green biosynthesis of 12β-O-Glc-PPD in *Saccharomyces cerevisiae*. Green Chem..

[22] Ahmad N., Xu K., Wang J.N., Li C. (2020). Novel catalytic glycosylation of Glycyrrhetinic acid by UDP-glycosyltransferases from *Bacillus subtilis*. Biochem. Eng. J..

[23] Hansen E.H., Moller B.L., Kock G.R., Bünner C.M., Kristensen C., Jensen O.R., Okkels F.T., Olsen C.E., Motawia M.S., Hansen J. (2009). De Novo Biosynthesis of Vanillin in Fission Yeast (*Schizosaccharomyces pombe*) and Baker’s Yeast (*Saccharomyces cerevisiae*). Appl. Environ. Microbiol..

[24] Gallage N.J., Hansen E.H., Kannangara R., Olsen C.E., Motawia M.S., Jorgensen K., Holme I., Hebelstrup K., Grisoni M., Moller B.L. (2014). Vanillin formation from ferulic acid in *Vanilla planifolia* is catalysed by a single enzyme. Nat. Commun..

[25] Rahmani B., Akbari H., Esmaeili H., Solaimanian S., Mobasheri M., Shiri-Shahsavar M.R. (2025). Discovering bioactive pharmaceuticals from natural products for type 2 diabetes mellitus using network pharmacology, molecular docking, and molecular dynamics. Sci. Rep..

[26] Jakhar R., Dangi M., Khichi A., Chhillar A.K. (2020). Relevance of Molecular Docking Studies in Drug Designing. Curr. Bioinform..

[27] Zhu J.T., Gu Z.H., Pei J.F., Lai L.H. (2024). DiffBindFR: An SE(3) equivariant network for flexible protein-ligand docking. Chem. Sci..

[28] Aijijiyah N.P., Wati F.A., Rahayu R., Srilistiani A., Mahzumi F., Aulia T., Santoso L., Pamela E., Ramadhani E.Y., Ilfahmi Y.A. (2023). Synthesis, *α*-glucosidase inhibitory activity, and molecular docking of cinnamamides. Med. Chem. Res..

[29] Jiang X., Zhang X.H., Li Y.X., Chen K., Lin B., Lu T.Q., Yang M., Chen G.T., Fan B.Y., Wang W.L. (2023). Chemical constituents from the leaves of *Cinnamomum camphora* and their *α*-glucosidase inhibitory activities. Phytochem. Lett..

[30] Rasouli H., Hosseini-Ghazvini S.M.B., Adibi H., Khodarahmi R. (2017). Differential *α*-amylase/*α*-glucosidase inhibitory activities of plant-derived phenolic compounds: A virtual screening perspective for the treatment of obesity and diabetes. Food Funct..

[31] Wei Y.L., Yu N., Wang Z.Y., Hao Y.M., Wang Z.W., Yang Z.H., Liu J., Wang J. (2022). Analysis of the multi-physiological and functional mechanism of wheat alkylresorcinols based on reverse molecular docking and network pharmacology. Food Funct..

[32] Luo W.F., Deng J., He J.C., Yin L., You R., Zhang L.K., Shen J., Han Z.P., Xie F.M., He J.H. (2023). Integration of molecular docking, molecular dynamics and network pharmacology to explore the multi-target pharmacology of fenugreek against diabetes. J. Cell. Mol. Med..

