# Supplementary material for: Efficient Synthesis of Glucovanillin and Elucidation of Its Molecular Mechanisms in Ameliorating T2DM via Core Target Modulation and α-Glucosidase Inhibition
Source: Molecules. 2026 Jun 24;31(13):2228. doi: 10.3390/molecules31132228 (PMC13362808; doi:10.3390/molecules31132228)
Supplement: Supplementary file 1 [file molecules-31-02228-s001.zip › molecules-4310284-supplementary.pdf]

Supplementary Materials

Efficient Synthesis of Glucovanillin and Elucidation of Its Molecular Mechanisms in Ameliorating T2DM via Core Target Modulation and α-Glucosidase Inhibition

Huanyu Zhang [1,†], Weiqian Zhang [1,†], Fangya Li [2], Xinyao Lu [1], Yuping Yan [1,*] and Dan Zhang [1,*]

[1] Traditional Chinese Medicine Processing Technology Innovation Center of Hebei Province, Hebei University of Chinese Medicine, Shijiazhuang 050200, China; zhanghychn@163.com (H.Z.); yjs20252134@hebcm.edu.cn (W.Z.); yjs20242112@hebcm.edu.cn (X.L.)
[2] School of Mathematical Sciences, Hebei Normal University, Shijiazhuang 050024, China; lifangya@hebtu.edu.cn
* Correspondence: yanyuping2008@126.com (Y.Y.); zhangdan@hebcm.edu.cn (D.Z.)
† These authors contributed equally to this work.

1. Supplementary Figures

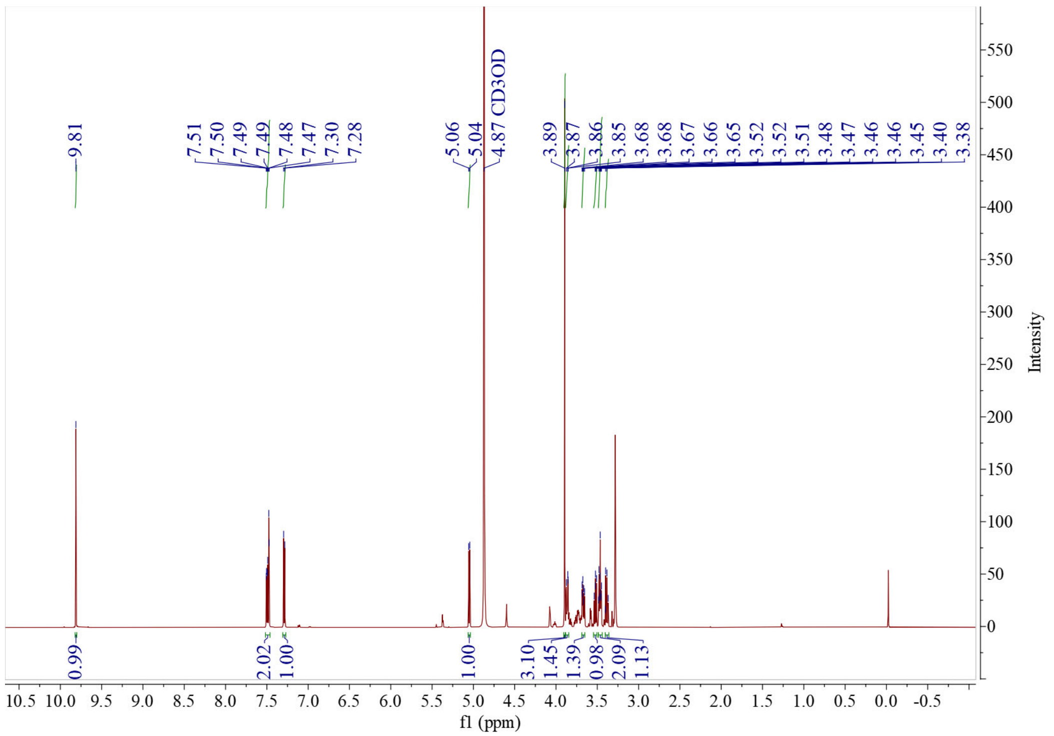

Figure S1. ^{1}H NMR spectrum of P3 in methanol–*d*4 (600 MHz).

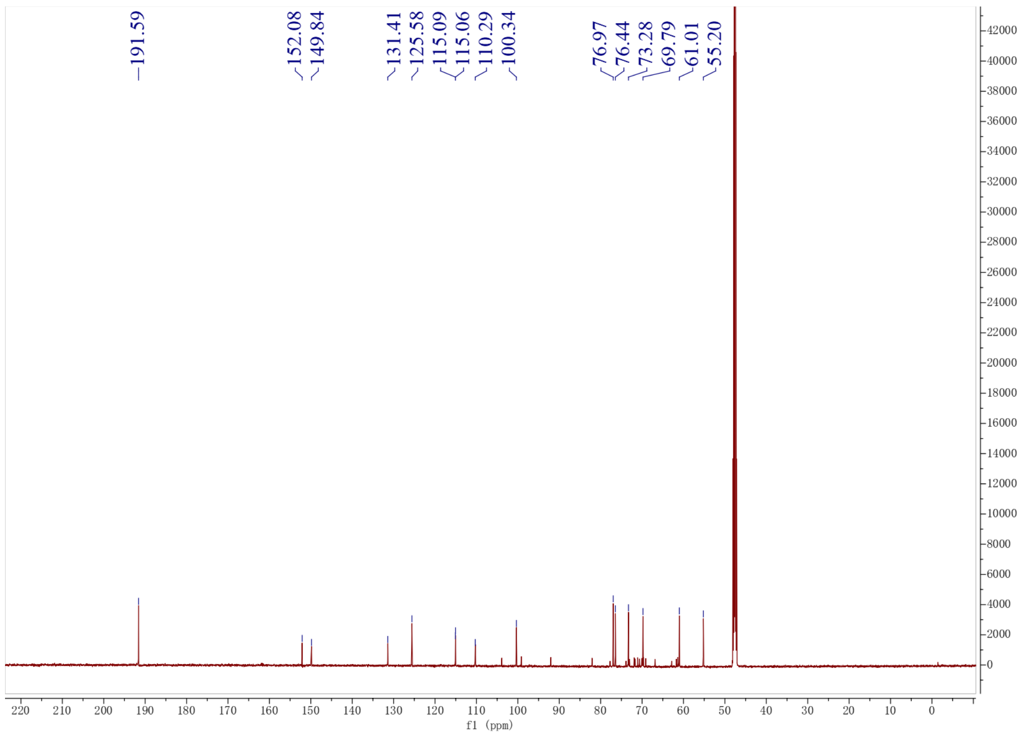

Figure S2. ^{13}C NMR spectrum of P3 in methanol–*d*4 (600 MHz).

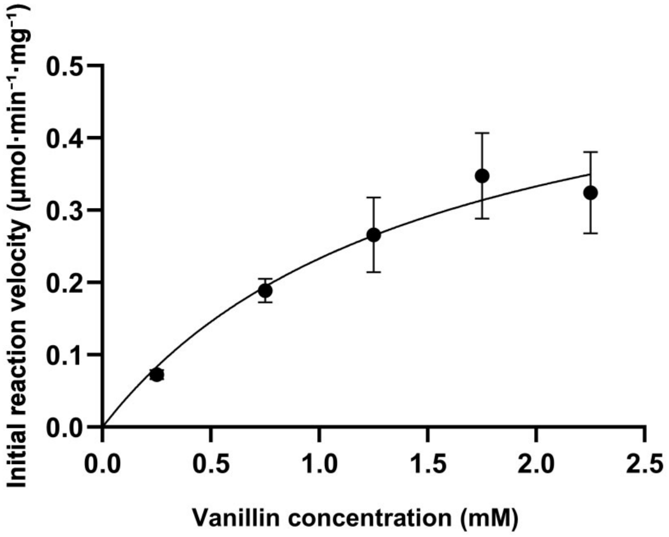

Figure S3. Enzyme kinetics of UGT109A1 toward vanillin.
